# Supplementary material for: Micronutrient Intake during Complementary Feeding in Very Low Birth Weight Infants Comparing Early and Late Introduction of Solid Foods: A Secondary Outcome Analysis
Source: Nutrients. 2024 Sep 27;16(19):3279. doi: 10.3390/nu16193279 (PMC11478718; doi:10.3390/nu16193279)
Supplement: Supplementary file 1 [file nutrients-16-03279-s001.zip › nutrients-3212742-supplementary.pdf]

Supplemental material – Timepoint of solid food introduction

Part II: Micronutrient Intake during Complementary Feeding in Very Low Birth Weight Infants comparing Early and Late Introduction of Solid Foods: A Secondary Outcome Analysis

1. Micronutrient intake comparing early and late introduction of solid foods

Nutrient intake was evaluated by comparing early and late introduction of solid foods using linear mixed-effects models. These models included the complementary feeding group, sex, gestational age, and nutrition at 6 weeks corrected age (CA) as covariates, with a random intercept to address potential correlation among siblings of multiple births. Marginal means, calculated from these models and averaged across covariates, were determined for the two groups. Standard errors and p-values were then calculated to test the null hypothesis of no difference between the groups. The p-values for between-group comparisons of the same nutrient at different timepoints were adjusted using the Bonferroni-Holm method. Statistical significance was set at p-adj. < 0.05.

The nutritional sources were categorized into two main types: solids and milk (breastmilk, infant formula). The distribution of these sources is presented as a percentage of the total nutritional content. It's important to note that tea, water, and liquid beverages were collectively categorized as solids, despite liquids not being considered in determining the beginning of complementary feeding.

Supplemental table S1: *Dietary micronutrient intake comparing early and late introduction of complementary feeding.*

CA	Dietary iron intake (mg/kg/d)							Dietary iron intake (mg/d)						
	Early			Late				Early			Late			
	Mean ± SE	% (milk)	% (solids)	Mean ± SE	% (milk)	% (solids)	p-adj.	Mean ± SE	% (milk)	% (solids)	Mean ± SE	% (milk)	% (solids)	p-adj.
6 weeks	0.72 (±0.60)	99.9	0.1	0.62 (±0.06)	100.0	0.0	0.32	3.05 (±0.23)	99.9	0.1	2.59 (±0.23)	100.0	0.0	0.18
12 weeks	0.64 (±0.04)	94.3	35.7	0.55 (±0.04)	99.8	0.2	0.28	3.49 (±0.20)	94.3	35.7	2.87 (±0.23)	99.8	0.2	0.09
6 months	0.71 (±0.03)	57.4	42.6	0.58 (±0.04)	64.9	35.1	**0.04**	4.89 (±0.21)	57.4	42.6	3.78 (±0.23)	64.9	35.1	**0.002**
9 months	0.74 (±0.04)	40.7	59.3	0 .68 (±0.04)	41.7	58.3	0.32	5.98 (±0.29)	40.7	59.3	5.16 (±0.31)	41.7	58.3	0.09
12 months	0.71 (±0.04)	39.1	60.9	0.75 (±0.04)	32.5	67.5	0.48	6.24 (±0.34)	39.1	60.9	6.28 (±0.34)	32.5	67.5	0.93
	/							Recommended daily iron intake: 0-3 months: 0.3 mg/d, 4-12 months: 11 mg/d.						

CA	Total iron intake (mg/kg/d) – dietary iron + supplementation							Dietary vitamin D intake (IU/d)						
	Early			Late				Early			Late			
	Mean ± SE	% (milk)	% (solids)	Mean ± SE	% (milk)	% (solids)	p-adj.	Mean ± SE	% (milk)	% (solids)	Mean ± SE	% (milk)	% (solids)	p-adj.
6 weeks	4.15 (±0.16)	/	/	4.33 (±0.16)	/	/	0.56	328 (±29)	99.9	0.1	275 (±29)	100.0	0.0	0.74
12 weeks	3.59 (±0.13)	/	/	3.62 (±0.15)	/	/	0.86	287 (±19)	99.5	0.5	270 (±21)	100.0	0.0	0.74
6 months	2.40 (±0.16)	/	/	2.59 (±0.18)	/	/	0.56	283 (±17)	81.9	18.1	260 (±19)	85.5	14.5	0.74
9 months	1.27 (±0.16)	/	/	1.55 (±0.17)	/	/	0.56	273 (±21)	70.0	30.0	285 (±23)	67.5	32.5	0.74
12 months	1.00 (±0.15)	/	/	1.48 (±0.16)	/	/	0.15	264 (±21)	63.3	36.7	255 (±21)	60.0	40.0	0.74
	Recommended daily iron supplementation: 2–3 mg/kg/d until 6–12 months of age.							/						

CA	Total vitamin D intake (IU/d) – dietary iron + supplementation							Dietary calcium intake (mg/d)						
	Early			Late				Early			Late			
	Mean ± SE	% (milk)	% (solids)	Mean ± SE	% (milk)	% (solids)	p-adj.	Mean ± SE	% (milk)	% (solids)	Mean ± SE	% (milk)	% (solids)	p-adj.
6 weeks	1116 (±39)	/	/	1011 (±39)	/	/	0.17	388 (±18)	99.9	0.1	345 (±18)	100.0	0.0	0.20
12 weeks	1016 (±28)	/	/	1012 (±32)	/	/	0.92	373 (±14)	97.3	2.7	254 (±15)	100.0	0.0	0.57
6 months	1021 (±26)	/	/	1011 (±29)	/	/	0.92	428 (±16)	72.8	27.2	386 (±17)	79.9	20.1	0.20
9 months	986 (±37)	/	/	993 (±36)	/	/	0.92	480 (±25)	52.3	47.7	506 (±26)	51.7	48.3	0.61
12 months	692 (±55)	/	/	833 (±57)	/	/	0.17	551 (±24)	43.7	56.3	537 (±24)	39.5	60.5	0.68
	Recommended daily vitamin D intake: 800-1000 IU/d.							Recommended daily calcium intake: 0-3 months: 220 mg/d, 4-12 months: 330 mg/d.						

CA	Dietary phosphorus intake (mg/d)							Dietary zinc intake (mg/d)						
	Early			Late				Early			Late			
	Mean ± SE	% (milk)	% (solids)	Mean ± SE	% (milk)	% (solids)	p-adj.	Mean ± SE	% (milk)	% (solids)	Mean ± SE	% (milk)	% (solids)	p-adj.
6 weeks	223 (±12)	99.9	0.1	205 (±12)	100.0	0.0	0.42	3.3 (±0.2)	99.9	0.1	2.8 (±0.2)	100.0	0.0	0.07
12 weeks	232 (±10)	94.7	5.2	210 (±11)	99.9	0.1	0.34	3.4 (±0.2)	97.7	2.3	2.8 (±0.2)	99.1	0.9	0.06
6 months	341 (±14)	55.9	44.1	286 (±15)	67.0	33.0	**0.04**	4.1 (±0.2)	64.6	35.4	3.5 (±0.2)	71.6	28.4	0.06
9 months	443 (±20)	35.6	64.4	442 (±20)	36.2	63.8	0.98	4.4 (±0.2)	45.8	54.2	4.4 (±0.2)	47.0	53.0	0.96
12 months	541 (±23)	29.7	70.3	511 (±23)	26.6	73.4	0.43	4.9 (±0.2)	37.1	62.9	4.9 (±0.2)	35.0	65.0	0.96
	Recommended daily phosphorus intake: 0-3 months: 120 mg/d, 4-12 months: 180 mg/d.							Recommended daily zinc intake: 0–3 months: 1.5 mg/d; 4–12 months: 2.5 mg/d.						

CA: corrected age; SE: standard error. p-adj. <0.05 were considered statistically significant and marked bold. % (milk, solids) provide the proportional intake of the respective nutrient that was provided either by solids or milk (human milk, formula)

2. Subgroup analysis: micronutrient intake in infants with and without comorbidities

Supplemental table S2: *Number of infants with neonatal comorbidities.*

Parameter	Early (n=115)	Late (n=82)
Necrotizing enterocolitis ≥ grade II	5 (4%)	6 (7%)
Bronchopulmonary dysplasia	14 (12%)	23 (28%)
Intraventricular haemorrhage ≥ grade II	17 (15%)	12 (15%)
Periventricular leukomalacia	0 (0%)	1 (1%)

2.1. Subgroup analysis of micronutrient intake in infants without comorbidities and infants with BPD, NEC or IVH

Nutrient intake was evaluated by comparing infants without comorbidities and infants diagnosed with bronchopulmonary dysplasia (BPD), necrotizing enterocolitis ≥ grade II (NEC), or intraventricular hemorrhage ≥ grade II (IVH). To detect differences between study subgroups, students t-test or Mann-Whitney-U-test were applied. Standard errors and adjusted p-values were calculated to test the null hypothesis of no difference between the groups. Statistical significance was set at p-adj. < 0.05. As an additional analysis, the p-values for between-subgroup comparisons of the same nutrient at different timepoints were adjusted using the Bonferroni-Holm method. The statistical analysis was performed using R Studio (Core Team, 2022).

Supplemental table S3: *Micronutrient intake in infants without comorbidities and infants with BPD, NEC or IVH.*

CA	Dietary iron intake (mg/kg/d)						
	Infants without BPD, NEC, IVH<br>Mean ± SE	BPD<br>Mean ± SE	p-adj.	NEC<br>Mean ± SE	p-adj.	IVH<br>Mean ± SE	p-adj.
6 weeks	0.72 (±0.05)	0.92 (±0.15)	0.98	0.77 (±0.20)	0.98	0.81 (±0.14)	0.98
12 weeks	0.64 (±0.04)	0.65 (±0.07)	0.98	0.55 (±0.13)	0.98	0.57 (±0.10)	0.98
6 months	0.63 (±0.03)	0.62 (±0.06)	0.98	0.68 (±0.10)	0.98	0.65 (±0.07)	0.98
9 months	0.68 (±0.03)	0.73 (±0.04)	0.98	0.64 (±0.08)	0.98	0.67 (±0.08)	0.98
12 months	0.73 (±0.03)	0.73 (±0.07)	0.98	0.64 (±0.05)	0.98	0.68 (±0.06)	0.98
			/				

CA	Total iron intake (mg/kg/d)						
	Infants without BPD, NEC, IVH<br>Mean ± SE	BPD<br>Mean ± SE	p-adj.	NEC<br>Mean ± SE	p-adj.	IVH<br>Mean ± SE	p-adj.
6 weeks	4.35 (±0.13)	4.51 (±0.24)	0.75	4.12 (±0.24)	0.84	3.61 (±0.17)	0.07
12 weeks	3.70 (±0.11)	3.60 (±0.17)	0.83	3.80 (±0.27)	0.84	3.27 (±0.19)	0.24
6 months	2.34 (±0.13)	2.54 (±0.22)	0.75	3.51 (±0.31)	0.05	2.44 (±0.30)	0.86
9 months	1.00 (±0.10)	1.82 (±0.29)	0.07	2.54 (±0.47)	0.05	1.67 (±0.18)	0.07
12 months	1.01 (±0.11)	1.51 (±0.22)	0.07	1.80 (±0.43)	0.10	1.46 (±0.26)	0.25

Recommended daily iron supplementation: 2–3 mg/kg/d until 6–12 months of age.

CA	Dietary vitamin D intake (IU/d)						
	Infants without BPD, NEC, IVH	BPD	p-adj.	NEC	p-adj.	IVH	p-adj.
	Mean ± SE	Mean ± SE		Mean ± SE		Mean ± SE	
6 weeks	354 (±22)	302 (±44)	0.94	388 (±92)	0.94	359 (±47)	0.94
12 weeks	330 (±19)	299 (±30)	0.94	364 (±68)	0.94	323 (±48)	0.94
6 months	282 (±17)	293 (±33)	0.94	296 (±55)	0.94	273 (±36)	0.94
9 months	283 (±20)	298 (±38)	0.94	293 (±71)	0.94	238 (±41)	0.94
12 months	266 (±18)	263 (±29)	0.94	284 (±43)	0.94	224 (±33)	0.94

/

CA	Total vitamin D intake (IU/d)						
	Infants without BPD, NEC, IVH	BPD	p-adj.	NEC	p-adj.	IVH	p-adj.
	Mean ± SE	Mean ± SE		Mean ± SE		Mean ± SE	
6 weeks	1118 (±26)	1072 (±62)	0.98	1106 (±136)	0.99	1176 (±78)	0.98
12 weeks	1068 (±26)	1069 (±47)	0.98	1106 (±90)	0.99	1054 (±64)	0.98
6 months	1045 (±18)	1048 (±60)	0.98	1021 (±79)	0.98	970 (±70)	0.98
9 months	1007 (±30)	1006 (±58)	0.99	1017 (±128)	0.98	1060 (±86)	0.98
12 months	797 (±44)	871 (±74)	0.98	740 (±146)	0.98	719 (±111)	0.98

Recommended daily vitamin D intake: 800-1000 IU/d.

CA	Dietary calcium intake (mg/d)						
	Infants without BPD, NEC, IVH	BPD	p-adj.	NEC	p-adj.	IVH	p-adj.
	Mean ± SE	Mean ± SE		Mean ± SE		Mean ± SE	
6 weeks	392 (±13)	388 (±37)	0.80	418 (±48)	0.80	380 (±44)	0.80
12 weeks	392 (±12)	378 (±26)	0.80	374 (±23)	0.87	368 (±37)	0.80
6 months	420 (±14)	406 (±25)	0.80	396 (±36)	0.80	396 (±30)	0.80
9 months	481 (±22)	504 (±31)	0.80	512 (±58)	0.80	483 (±38)	0.80
12 months	560 (±19)	532 (±35)	0.80	477 (±35)	0.80	496 (±35)	0.80

Recommended daily calcium intake: 0-3 months: 220 mg/d, 4-12 months: 330 mg/d.

CA	Dietary phosphorus intake (mg/d)						
	Infants without BPD, NEC, IVH	BPD	p-adj.	NEC	p-adj.	IVH	p-adj.
	Mean ± SE	Mean ± SE		Mean ± SE		Mean ± SE	
6 weeks	236 (±9)	225 (±22)	0.52	243 (±31)	0.76	220 (±27)	0.62
12 weeks	244 (±9)	226 (±17)	0.56	210 (±14)	0.56	219 (±24)	0.52
6 months	337 (±14)	295 (±18)	0.52	306 (±33)	0.60	295 (±22)	0.52
9 months	451 (±16)	439 (±21)	0.97	380 (±53)	0.51	392 (±36)	0.37
12 months	548 (±17)	493 (±36)	0.37	397 (±35)	**0.04**	462 (±34)	0.10

Recommended daily phosphorus intake: 0-3 months: 120 mg/d, 4-12 months: 180 mg/d.

CA	Dietary zinc intake (mg/d)						
	Infants without BPD, NEC, IVH	BPD	p-adj.	NEC	p-adj.	IVH	p-adj.
	Mean ± SE	Mean ± SE		Mean ± SE		Mean ± SE	
6 weeks	3.42 (±0.15)	3.56 (±0.46)	0.95	3.44 (±0.77)	0.95	3.95 (±0.55)	0.95
12 weeks	3.40 (±0.15)	3.58 (±0.14)	0.95	3.04 (±0.46)	0.95	3.48 (±0.48)	0.95
6 months	3.87 (±0.16)	3.90 (±0.30)	0.95	3.83 (±0.59)	0.95	3.79 (±0.38)	0.95
9 months	4.42 (±0.16)	4.22 (±0.25)	0.95	5.04 (±0.59)	0.95	4.06 (±0.45)	0.95
12 months	5.08 (±0.15)	4.67 (±0.24)	0.95	4.67 (±0.51)	0.95	4.45 (±0.38)	0.95

Recommended daily zinc intake: 0–3 months: 1.5 mg/d; 4–12 months: 2.5 mg/d.
CA: corrected age; BPD: bronchopulmonary dysplasia; NEC: necrotizing enterocolitis;, IVH: intraventricular hemorrhage. SE: standard error. p-adj. <0.05 were considered statistically significant and marked bold.

2.2. Subgroup analysis: micronutrient intake comparing early and late introduction of solid foods in infants with and without comorbidities

Nutrient intake was evaluated by comparing the timepoint of solid food introduction (early vs. late) in infants without comorbidities and infants diagnosed with bronchopulmonary dysplasia (BPD), necrotizing enterocolitis ≥ grade II (NEC), or intraventricular hemorrhage ≥ grade II (IVH). To detect differences between study subgroups, students t-test or Mann-Whitney-U-test were applied. For infants with NEC no statistical testing was conducted between the early and late group due to low the low number of infants with NEC. Standard errors and adjusted p-values were calculated to test the null hypothesis of no difference between the groups. Statistical significance was set at p-adj. < 0.05. As an additional analysis, the p-values for between-subgroup comparisons of the same nutrient at different timepoints were adjusted using the Bonferroni-Holm method. The statistical analysis was performed using R Studio (Core Team, 2022).

2.2.1. Micronutrient intake comparing early and late introduction of solids in infants with BPD

Supplemental table S4: *Numbers of valid dietary records from infants with/without bronchopulmonary dysplasia.*

CA	EARLY			LATE		
	total	Infants without BPD, NEC, IVH	BPD	total	Infants without BPD, NEC, IVH	BPD
**6 weeks**	75	64 (85%)	11 (15%)	54	37 (69%)	17 (31%)
**12 weeks**	92	79 (86%)	13 (14%)	57	36 (63%)	21 (37%)
**6 months**	77	66 (86%)	11 (14%)	58	41 (71%)	17 (29%)
**9 months**	54	48 (89%)	6 (11%)	46	29 (65%)	16 (35%)
**12 months**	53	48 (91%)	5 (9%)	49	32 (65%)	17 (35%)

CA: corrected age; BPD: bronchopulmonary dysplasia; NEC: necrotizing enterocolitis; IVH: intraventricular hemorrhage. Data are presented as numbers with percentages in parentheses. Not all dietary records included information on vitamin D and iron supplementation. Consequently, the number of available results for total vitamin D and iron varies from the stated numbers

Supplemental table S5: *Micronutrient intake comparing early and late introduction of solids in infants with bronchopulmonary dysplasia.*

CA	Dietary iron intake (mg/kg/d)			Total iron intake (mg/kg/d)		
	**Early Mean ± SE**	**Late Mean ± SE**	**p-adj.**	**Early Mean ± SE**	**Late Mean ± SE**	**p-adj.**
**6 weeks**	1.12 (±0.25)	0.76 (±0.17)	0.35	4.30 (±0.42)	4.65 (±0.29)	0.69
**12 weeks**	0.81 (±0.14)	0.55 (±0.07)	0.21	3.47 (±0.27)	3.68 (±0.22)	0.69
**6 months**	0.80 (±0.08)	0.50 (±0.07)	0.07	3.13 (±0.24)	2.16 (±0.29)	0.13
**9 months**	0.78 (±0.11)	0.71(±0.05)	0.59	2.22 (±0.54)	1.65 (±0.35)	0.69
**12 months**	0.76 (±0.11)	0.72 (±0.08)	0.67	1.39 (±0.42)	1.56 (±0.27)	0.72
**CA**	**Dietary vitamin D intake (IU/d)**			**Total vitamin D intake (IU/d)**		
	**Early Mean ± SE**	**Late Mean ± SE**	**p-adj.**	**Early Mean ± SE**	**Late Mean ± SE**	**p-adj.**
**6 weeks**	339 (±58)	278 (±63)	0.57	1117 (±111)	1042 (±74)	0.87
**12 weeks**	368 (±50)	256 (±34)	0.16	1139 (±97)	1026 (±46)	0.68
**6 months**	368 (±55)	244 (±37)	0.16	1132 (±125)	992 (±56)	0.74
**9 months**	250 (±70)	316 (±46)	0.57	927 (±88)	1039 (±73)	0.68
**12 months**	276 (±75)	259 (±32)	0.82	777 (±185)	898 (±81)	0.68
**CA**	**Dietary calcium intake (mg/d)**			**Dietary phosphorus intake (mg/d)**		
	**Early Mean ± SE**	**Late Mean ± SE**	**p-adj.**	**Early Mean ± SE**	**Late Mean ± SE**	**p-adj.**
**6 weeks**	444 (±67)	351 (±41)	0.32	251 (±38)	207 (±28)	0.42
**12 weeks**	447 (±53)	335 (±22)	0.17	273 (±34)	198 (±14)	0.13
**6 months**	494 (±42)	348 (±23)	**0.03**	357 (±29)	254 (±18)	**0.02**
**9 months**	524 (±82)	495 (±31)	0.69	485 (±41)	422 (±23)	0.30
**12 months**	637 (±88)	501 (±36)	0.18	571 (±93)	470 (±37)	0.30
**CA**	**Dietary zinc intake (mg/d)**			CA: corrected age; SE: standard error. p-adj. <0.05 were considered statistically significant and marked bold.		
	**Early Mean ± SE**	**Late Mean ± SE**	**p-adj.**			
**6 weeks**	4.20 (±0.89)	3.16 (±0.50)	0.39			
**12 weeks**	4.35 (±0.65)	3.10 (±0.33)	0.18			
**6 months**	4.72 (±0.57)	3.36 (±0.28)	0.13			
**9 months**	4.10 (±0.47)	4.26 (±0.30)	0.80			
**12 months**	5.12 (±0.64)	4.54 (±0.24)	0.39			

2.2.2. Micronutrient intake comparing early and late introduction of solids in infants with NEC

Supplemental table S6: *Numbers of valid dietary records from infants with/without necrotizing enterocolitis ≥ grade II.*

CA	EARLY			LATE		
	total	Infants without BPD, NEC, IVH	NEC	total	Infants without BPD, NEC, IVH	NEC
6 weeks	67	64 (85%)	3 (5%)	43	37 (86%)	6 (14%)
12 weeks	84	79 (94%)	5 (6%)	42	36 (86%)	6 (14%)
6 months	70	66 (94%)	4 (6%)	47	41 (85%)	6 (15%)
9 months	50	48 (96%)	2 (4%)	33	29 (88%)	4 (12%)
12 months	52	48 (92%)	4 (8%)	37	32 (86%)	5 (14%)

CA: corrected age; BPD: bronchopulmonary dysplasia; NEC: necrotizing enterocolitis; IVH: intraventricular hemorrhage. Data are presented as numbers with percentages in parentheses. Not all dietary records included information on vitamin D and iron supplementation. Consequently, the number of available results for total vitamin D and iron varies from the stated numbers.

Supplemental table S7: *Micronutrient intake comparing early and late introduction of solids in infants with necrotizing enterocolitis ≥ grade II.*

CA	Dietary iron intake (mg/kg/d)		Total iron intake (mg/kg/d)	
	Early	Late	Early	Late
	Mean ± SE	Mean ± SE	Mean ± SE	Mean ± SE
6 weeks	0.66 (±0.40)	0.82 (±0.25)	4.52 (±0.25)	3.91 (±0.32)
12 weeks	0.50 (±0.22)	0.60 (±0.18)	4.15 (±0.42)	3.51 (±0.32)
6 months	0.83 (±0.20)	0.58 (±0.11)	3.68 (±0.40)	3.40 (±0.48)
9 months	0.75 (±0.04)	0.59 (±0.12)	3.42 (±NA)	2.25 (±0.51)
12 months	0.68 (±0.08)	0.61 (±0.07)	1.90 (±0.63)	1.71 (±0.65)

CA	Dietary vitamin D intake (IU/d)		Total vitamin D intake (IU/d)	
	Early	Late	Early	Late
	Mean ± SE	Mean ± SE	Mean ± SE	Mean ± SE
6 weeks	538 (±194)	313 (±96)	1289 (±146)	1015 (±181)
12 weeks	390 (±111)	342 (±93)	1102 (±116)	1110 (±143)
6 months	348 (±92)	261 (±72)	740 (±105)	987 (±117)
9 months	458 (±17)	210 (±75)	1241 (±NA)	942 (±147)
12 months	288 (±63)	280 (±66)	688 (±214)	780 (±220)

CA	Dietary calcium intake (mg/d)		Dietary phosphorus intake (mg/d)	
	Early	Late	Early	Late
	Mean ± SE	Mean ± SE	Mean ± SE	Mean ± SE
6 weeks	406 (±66)	422 (±69)	244 (±42)	242 (±44)
12 weeks	365 (±32)	381 (±36)	209 (±19)	210 (±23)
6 months	434 (±48)	371 (±51)	273 (±67)	262 (±21)
9 months	450 (±7)	543 (±86)	363 (±84)	388 (±75)
12 months	412 (±30)	529 (±47)	386 (±70)	406 (±36)

CA	Dietary zinc intake (mg/d)	
	Early	Late
	Mean ± SE	Mean ± SE
6 weeks	2.32(±1.19)	4.00 (±0.97)
12 weeks	2.65 (±0.79)	3.38 (±0.57)
6 months	3.92 (±1.31)	3.77 (±0.58)
9 months	5.81 (±0.94)	4.67 (±0.77)
12 months	4.86 (±0.61)	4.53 (±0.82)

CA: corrected age; SE: standard error. No p-values were calculated between the groups due to the small number of infants with necrotizing enterocolitis.

2.2.3. Micronutrient intake comparing early and late introduction of solids in infants with IVH

Supplemental table S8: *Numbers of valid dietary records from infants with/without intraventricular hemorrhage ≥ grade II.*

CA	EARLY			LATE		
	total	Infants without BPD, NEC, IVH	IVH	total	Infants without BPD, NEC, IVH	IVH
**6 weeks**	75	64 (85%)	11 (15%)	42	37 (88%)	5 (12%)
**12 weeks**	91	79 (87%)	12 (13%)	44	36 (82%)	8 (18%)
**6 months**	78	66 (85%)	12 (15%)	49	41 (84%)	8 (16%)
**9 months**	56	48 (86%)	8 (14%)	36	29 (81%)	7 (19%)
**12 months**	59	48 (86%)	11 (14%)	41	32 (78%)	9 (22%)

CA: corrected age; BPD: bronchopulmonary dysplasia; NEC: necrotizing enterocolitis; IVH: intraventricular hemorrhage. Data are presented as numbers with percentages in parentheses. Not all dietary records included information on vitamin D and iron supplementation. Consequently, the number of available results for total vitamin D and iron varies from the stated numbers.

Supplemental table S9: *Micronutrient intake comparing early and late introduction of solids in infants with intraventricular hemorrhage ≥ grade II.*

CA	Dietary iron intake (mg/kg/d)			Total iron intake (mg/kg/d)		
	Early<br>Mean ± SE	Late<br>Mean ± SE	p-adj.	Early<br>Mean ± SE	Late<br>Mean ± SE	p-adj.
**6 weeks**	0.90 (±0.16)	0.59 (±0.26)	0.30	3.56 (±0.19)	3.73 (±0.36)	0.71
**12 weeks**	0.68 (±0.13)	0.39 (±0.12)	0.28	3.21 (±0.28)	3.35 (±0.23)	0.71
**6 months**	0.72 (±0.10)	0.53 (±0.10)	0.28	2.58 (±0.39)	2.26 (±0.50)	0.71
**9 months**	0.77 (±0.12)	0.56 (±0.09)	0.28	1.18 (±0.33)	2.07 (±0.39)	0.61
**12 months**	0.77 (±0.09)	0.57 (±0.05)	0.28	1.26 (±0.31)	1.76 (±0.47)	0.71

CA	Dietary vitamin D intake (IU/d)			Total vitamin D intake (IU/d)		
	Early<br>Mean ± SE	Late<br>Mean ± SE	p-adj.	Early<br>Mean ± SE	Late<br>Mean ± SE	p-adj.
**6 weeks**	384 (±46)	304 (±119)	0.93	1213 (±101)	1096 (±179)	0.80
**12 weeks**	357 (±60)	273 (±82)	0.93	1071 (±74)	1030 (±122)	0.80
**6 months**	268 (±44)	289 (±67)	0.93	911 (±103)	1052 (±88)	0.80
**9 months**	239 (±45)	237 (±76)	0.98	1079 (±105)	1041 (±101)	0.80
**12 months**	242 (±40)	202 (±57)	0.93	725 (±167)	714 (±148)	0.80

CA	Dietary calcium intake (mg/d)			Dietary phosphorus intake (mg/d)		
	Early<br>Mean ± SE	Late<br>Mean ± SE	p-adj.	Early<br>Mean ± SE	Late<br>Mean ± SE	p-adj.
**6 weeks**	399 (±54)	338 (±81)	0.76	230 (±33)	199 (±50)	0.61
**12 weeks**	406 (±57)	311 (±31)	0.76	246 (±35)	181 (±21)	0.61
**6 months**	404 (±40)	385 (±48)	0.76	305 (±33)	279 (±26)	0.61
**9 months**	501 (±51)	462 (±61)	0.76	423 (±58)	358 (±40)	0.61
**12 months**	519 (±57)	470 (±38)	0.76	487 (±60)	433 (±22)	0.61

CA	Dietary zinc intake (mg/d)		
	Early<br>Mean ± SE	Late<br>Mean ± SE	p-adj.
**6 weeks**	4.33 (±0.64)	3.12 (±1.04)	0.53
**12 weeks**	4.15 (±0.69)	2.48 (±0.42)	0.11
**6 months**	4.01 (±0.51)	3.47 (±0.57)	0.61
**9 months**	4.30 (±0.60)	3.81 (±0.72)	0.61
**12 months**	4.93 (±0.54)	3.88 (±0.49)	0.44

CA: corrected age; SE: standard error. p-adj. <0.05 were considered statistically significant and marked bold.

3. Subgroup analysis: micronutrient intake in breastfed, formula-fed, mixed-fed infants and infants without milk-feeding

Nutrient intake was assessed by comparing breastfed with formula-fed, mixed-fed and infants who received neither breastmilk nor formula (=no milk). To detect differences between the type of feeding, students t-test or Mann-Whitney-U-test were applied (breastfed vs. formula, mixed, none). Statistical testing was not performed for mixed fed infants at 9 and 12 months CA and "no milk" from 6 weeks – 6 months CA, due to the small sample size. Standard errors and adjusted p-values were calculated to test the null hypothesis of no difference between the type of feeding. The p-values for between-type of feeding comparisons of the same nutrient at different timepoints were adjusted using the Bonferroni-Holm method. Statistical significance was set at p-adj. < 0.05. The statistical analysis was performed using R Studio (Core Team, 2022).

Supplemental table S10*: Micronutrient intake comparing breastfed, formula-fed, mixed-fed and no milk intake.*

**CA**	**Dietary iron intake (mg/kg/d)**						
	**Breastfed**	**Formula**	**p-adj.**	**Mixed**	**p-adj.**	**No milk**	**p-adj.**
	**Mean ± SE**	**Mean ± SE**		**Mean ± SE**		**Mean ± SE**	
**6 weeks**	0.17 (±0.03)	0.99 (±0.08)	**<0.001**	0.87 (±0.09)	**<0.001**		
**12 weeks**	0.09 (±0.00)	0.82 (±0.03)	**<0.001**	0.60 (±0.06)	**<0.001**	/	
**6 months**	0.26 (±0.03)	0.77 (±0.03)	**<0.001**	0.55 (±0.06)	**0.02**		
**9 months**	0.37 (±0.03)	0.77 (±0.03)	**<0.001**	0.89 (±0.31)	/	0.61 (±0.12)	0.04
**12 months**	0.52 (±0.05)	0.79 (±0.03)	**0.004**	0.60 (±0.06)	/	0.56 (±0.10)	0.69

/

**CA**	**Total iron intake (mg/kg/d)**						
	**Breastfed**	**Formula**	**p-adj.**	**Mixed**	**p-adj.**	**No milk**	**p-adj.**
	**Mean ± SE**	**Mean ± SE**		**Mean ± SE**		**Mean ± SE**	
**6 weeks**	3.72 (±0.15)	4.49 (±0.18)	**0.003**	4.62 (±0.28)	**0.007**		
**12 weeks**	3.51 (±0.21)	3.76 (±0.10)	0.46	3.48 (±0.19)	0.88	/	
**6 months**	2.44 (±0.24)	2.42 (±0.13)	0.98	2.44 (±0.28)	0.97		
**9 months**	0.65 (±0.13)	1.39 (±0.13)	**<0.001**	0.89 (±0.31)	/	2.11 (±0.48)	**0.007**
**12 months**	1.11 (±0.35)	1.24 (±0.12)	0.33	1.41 (±0.78)	/	1.56 (±0.33)	0.88

Recommended daily iron supplementation: 2–3 mg/kg/d until 6–12 months of age.

**CA**	**Dietary vitamin D intake (IU/d)**						
	**Breastfed**	**Formula**	**p-adj.**	**Mixed**	**p-adj.**	**No milk**	**p-adj.**
	**Mean ± SE**	**Mean ± SE**		**Mean ± SE**		**Mean ± SE**	
**6 weeks**	199 (±51)	419 (±16)	**<0.001**	307 (±36)	**0.002**		
**12 weeks**	68 (±28)	435 (±12)	**<0.001**	244 (±18)	**<0.001**	/	
**6 months**	54 (±8)	361 (±13)	**<0.001**	263 (±31)	**<0.001**		
**9 months**	68 (±19)	343 (±15)	**<0.001**	310 (±206)	/	150 (±60)	0.26
**12 months**	97 (±27)	313 (±14)	**<0.001**	371 (±79)	/	221 (±45)	0.21

/

**CA**	**Total vitamin D intake (IU/d)**						
	**Breastfed**	**Formula**	**p-adj.**	**Mixed**	**p-adj.**	**No milk**	**p-adj.**
	**Mean ± SE**	**Mean ± SE**		**Mean ± SE**		**Mean ± SE**	
**6 weeks**	960 (±57)	1196 (±35)	**<0.001**	1053 (±44)	**0.21**		
**12 weeks**	811 (±44)	1179 (±24)	**<0.001**	986 (±24)	**<0.001**	/	
**6 months**	809 (±26)	1124 (±19)	**<0.001**	984 (±57)	**0.008**		
**9 months**	799 (±34)	1084 (±23)	**<0.001**	794 (±226)	/	883 (±92)	0.51
**12 months**	714 (±98)	846 (±44)	0.11	806 (±167)	/	1010 (±58)	0.94

Recommended daily vitamin D intake: 800-1000 IU/d.

**CA**	**Dietary calcium intake (mg/d)**						
	**Breastfed**	**Formula**	**p-adj.**	**Mixed**	**p-adj.**	**No milk**	**p-adj.**
	**Mean ± SE**	**Mean ± SE**		**Mean ± SE**		**Mean ± SE**	
**6 weeks**	281 (±18)	438 (±24)	**<0.001**	387 (±23)	**<0.001**		
**12 weeks**	242 (±9)	444 (±13)	**<0.001**	352 (±14)	**<0.001**	/	
**6 months**	302 (±11)	449 (±14)	**<0.001**	428 (±28)	**<0.001**		
**9 months**	349 (±19)	513 (±18)	**<0.001**	593 (±207)	/	540 (±67)	**0.04**
**12 months**	445 (±34)	557 (±18)	**0.04**	563 (±42)	/	423 (±34)	0.15

Recommended daily calcium intake: 0-3 months: 220 mg/d, 4-12 months: 330 mg/d.

CA	Dietary phosphorus intake (mg/d)						
	Breastfed	Formula	p-adj.	Mixed	p-adj.	No milk	p-adj.
	Mean ± SE	Mean ± SE		Mean ± SE		Mean ± SE	
**6 weeks**	151 (±11)	267 (±14)	**<0.001**	227 (±13)	**<0.001**	/	
**12 weeks**	129 (±6)	282 (±9)	**<0.001**	205 (±44)	**<0.001**		
**6 months**	227 (±13)	360 (±12)	**<0.001**	317 (±27)	**0.004**		
**9 months**	342 (±16)	458 (±15)	**<0.001**	511 (±127)	/	515 (±68)	0.03
**12 months**	493 (±36)	531 (±18)	0.61	469 (±70)	/	411 (±29)	0.22
**Recommended daily phosphorus intake: 0-3 months: 120 mg/d, 4-12 months: 180 mg/d.**							
**CA**	**Dietary zinc intake (mg/d)**						
	**Breastfed**	**Formula**	**p-adj.**	**Mixed**	**p-adj.**	**No milk**	**p-adj.**
	**Mean ± SE**	**Mean ± SE**		**Mean ± SE**		**Mean ± SE**	
**6 weeks**	1.71 (±0.19)	4.30 (±0.28)	**<0.001**	3.27 (±0.32)	**<0.001**	/	
**12 weeks**	1.21 (±0.09)	4.30 (±0.13)	**<0.001**	2.89 (±0.19)	**<0.001**		
**6 months**	1.91 (±0.12)	4.52 (±0.14)	**<0.001**	3.79 (±0.31)	**<0.001**		
**9 months**	2.62 (±0.17)	4.82 (±0.12)	**<0.001**	5.06 (±1.48)	/	3.82 (±0.53)	0.04
**12 months**	4.05 (±0.32)	5.24 (±0.14)	**0.006**	5.24 (±0.71)	/	3.63 (±0.33)	0.72
**Recommended daily zinc intake: 0–3 months: 1.5 mg/d; 4–12 months: 2.5 mg/d.**							
CA: corrected age; SE: standard error. p-adj. <0.05 were considered statistically significant and marked bold. No milk: infants that received neither human milk nor infant formula.							

3.1. Micronutrient intake comparing early and late introduction of solid foods in breastfed, formula-fed, mixed-fed infants and infants without milk-feeding

Nutrient intake was evaluated by comparing the timepoint of solid food introduction (early vs. late) in breastfed, formula-fed, mixed-fed and infants who received neither breastmilk nor formula (=no milk). To detect differences between study subgroups, students t-test or Mann-Whitney-U-test were applied. Statistical testing was not performed for breastfed infants at 12 months CA, mixed fed infants at 9 and 12 months CA and "no milk fed" at 6 weeks - 9 months CA, due to small sample size. Standard errors and adjusted p-values were calculated to test the null hypothesis of no difference between the groups. The p-values for between-type of feeding comparisons of the same nutrient at different timepoints were adjusted using the Bonferroni-Holm method. Statistical significance was set at p-adj. < 0.05. The statistical analysis was performed using R Studio (Core Team, 2022).

Supplemental table S11*: Numbers of valid protocols according to type of feeding in the early and late group.*

CA	EARLY					LATE				
	**total**	**breastfed**	**formula**	**mixed**	**no milk**	**total**	**breastfed**	**formula**	**mixed**	**no milk**
6 weeks	86	15 (17%)	62 (72%)	9 (11%)	0 (%)	60	23 (38%)	27 (45%)	10 (17%)	0 (%)
12 weeks	106	18 (17%)	75 (71%)	13 (12%)	0 (%)	64	18 (28%)	31 (49%)	15 (23%)	0 (%)
6 months	90	17 (19%)	66 (73%)	7 (8%)	0 (%)	66	18 (27%)	37 (56%)	11 (17%)	0 (%)
9 months	63	11 (17%)	48 (76%)	1 (2%)	3 (5%)	51	11 (21%)	35 (69%)	2 (4%)	3 (6%)
12 months	65	3 (5%)	49 (75%)	1 (2%)	12 (18%)	57	8 (14%)	35 (61%)	2 (4%)	12 (21%)

Data are presented as numbers with percentages in parentheses. Mixed: breastmilk + formula. No milk: infants that received neither human milk nor infant formula. Not all dietary records included information on vitamin D and iron supplementation. Consequently, the number of available results for total vitamin D and iron varies from the stated numbers.

Supplemental table S12: *Micronutrient intake comparing early and late introduction of solid foods in breastfed, formula-fed, mixed-fed infants and without milk-feeding.*

CA	Dietary iron intake (mg/kg/d)											
	Breastfed			Formula			Mixed			No milk		
	Early	Late	p-adj.	Early	Late	p-adj.	Early	Late	p-adj.	Early	Late	p-adj.
	Mean ± SE	Mean ± SE		Mean ± SE	Mean ± SE		Mean ± SE	Mean ± SE		Mean ± SE	Mean ± SE	
**6 weeks**	0.24 (±0.06)	0.13 (±0.02)	0.22	1.03 (±0.06)	0.90 (±0.10)	0.29	0.82 (±0.13)	0.92 (±0.13)	0.72			
**12 weeks**	0.11 (±0.00)	0.08 (±0.00)	**0.04**	0.85 (±0.04)	0.74 (±0.05)	0.24	0.58 (±0.08)	0.62 (±0.09)	0.94	/		
**6 months**	0.30 (±0.04)	0.23 (±0.04)	0.40	0.82 (±0.04)	0.67 (±0.04)	0.24	0.65 (±0.11)	0.49 (±0.06)	0.36			
**9 months**	0.33 (±0.04)	0.41 (±0.05)	0.38	0.80 (±0.04)	0.72 (±0.04)	0.36	0.69 (±/)	1.00 (±0.51)	/	0.83 (±0.11)	0.38 (±0.08)	/
**12 months**	0.31 (±0.06)	0.60 (±0.04)	/	0.78 (±0.04)	0.80 (±0.05)	0.89	0.72 (±/)	0.54 (±0.00)	/	0.58 (±0.09)	0.63 (±0.09)	0.54

CA	Total iron intake (mg/kg/d)											
	Breastfed			Formula			Mixed			No milk		
	Early	Late	p-adj.	Early	Late	p-adj.	Early	Late	p-adj.	Early	Late	p-adj.
	Mean ± SE	Mean ± SE		Mean ± SE	Mean ± SE		Mean ± SE	Mean ± SE		Mean ± SE	Mean ± SE	
**6 weeks**	3.37 (±0.19)	3.72 (±0.23)	0.87	4.44 (±0.17)	4.60 (±0.19)	0.87	4.48 (±0.42)	4.76 (±0.40)	0.87			
**12 weeks**	3.68 (±0.25)	3.33 (±0.35)	0.87	3.80 (±0.13)	3.66 (±0.18)	0.87	3.27 (±0.21)	3.67 (±0.32)	0.87	/		
**6 months**	2.42 (±0.35)	2.46 (±0.35)	0.87	2.32 (±0.16)	2.62 (±0.20)	0.87	2.42 (±0.41)	2.47 (±0.41)	0.93			
**9 months**	0.34 (±0.06)	0.88 (±0.23)	0.21	1.34 (±0.14)	1.47 (±0.25)	0.87	0.69 (±/)	1.00 (±0.51)	/	1.67 (±0.81)	2.54 (±0.53)	/
**12 months**	0.32 (±0.06)	1.45 (±0.44)	/	1.20 (±0.13)	1.31 (±0.24)	0.87	0.72 (±/)	1.75 (±1.20)	/	0.61 (±0.10)	1.68 (±0.37)	0.21

CA	Dietary vitamin D intake (IU/d)											
	Breastfed			Formula			Mixed			No milk		
	Early	Late	p-adj.	Early	Late	p-adj.	Early	Late	p-adj.	Early	Late	p-adj.
	Mean ± SE	Mean ± SE		Mean ± SE	Mean ± SE		Mean ± SE	Mean ± SE		Mean ± SE	Mean ± SE	
**6 weeks**	247 (±95)	168 (±59)	0.60	420 (±15)	414 (±34)	0.89	397 (±57)	226 (±32)	0.15			
**12 weeks**	75 (±42)	60 (±39)	0.89	435 (±14)	433 (±24)	0.15	247 (±25)	242 (±25)	0.97	/		
**6 months**	68 (±14)	41 (±7)	0.15	367 (±15)	349 (±22)	0.89	270 (±43)	259 (±45)	0.97			
**9 months**	56 (±17)	80 (±20)	0.81	345 (±20)	341 (±20)	0.97	100 (±/)	416 (±307)	/	227 (±94)	74 (±56)	/
**12 months**	78 (±51)	105 (±33)	/	303 (±21)	329 (±20)	0.55	503 (±/)	306 (±78)	/	143 (±33)	126 (±21)	1.00

CA	Total vitamin D intake (IU/d)											
	Breastfed			Formula			Mixed			No milk		
	Early	Late	p-adj.	Early	Late	p-adj.	Early	Late	p-adj.	Early	Late	p-adj.
	Mean ± SE	Mean ± SE		Mean ± SE	Mean ± SE		Mean ± SE	Mean ± SE		Mean ± SE	Mean ± SE	
**6 weeks**	1068 (±100)	885 (±64)	0.31	1195 (±33)	1201 (±42)	0.76	1180 (±58)	937 (±41)	0.02			
**12 weeks**	817 (±40)	805 (±79)	0.76	1171 (±30)	1196 (±38)	0.76	1002 (±29)	974 (±37)	0.76	/		
**6 months**	801 (±52)	816 (±13)	0.34	1125 (±26)	1120 (±29)	0.76	1006 (±43)	969 (±94)	0.76			
**9 months**	834 (±41)	775 (±99)	0.76	1066 (±34)	1108 (±29)	0.76	900 (±/)	742 (±381)	/	1047 (±84)	775 (±106)	/
**12 months**	611 (±216)	759 (±113)	/	797 (±60)	921 (±64)	0.31	903 (±/)	758 (±278)	/	512 (±130)	755 (±92)	0.70

CA	Dietary calcium intake (mg/d)											
	Breastfed			Formula			Mixed			No milk		
	Early	Late	p-adj.	Early	Late	p-adj.	Early	Late	p-adj.	Early	Late	p-adj.
	Mean ± SE	Mean ± SE		Mean ± SE	Mean ± SE		Mean ± SE	Mean ± SE		Mean ± SE	Mean ± SE	
**6 weeks**	312 (±38)	261 (±17)	0.27	454 (±18)	401 (±27)	0.31	385 (±19)	389 (±42)	0.94			
**12 weeks**	251 (±15)	234 (±11)	0.17	456 (±15)	416 (±25)	0.27	332 (±16)	368 (±22)	0.45	/		
**6 months**	331 (±14)	276 (±14)	0.13	468 (±18)	416 (±22)	0.17	472 (±60)	400 (±26)	0.42			

**9 months**	341 (±39)	356 (±39)	0.74	507 (±23)	524 (±30)	0.72	575 (±/)	603 (±358)	/	587 (±50)	494 (±133)	/
**12 months**	403 (±93)	461 (±35)	/	564 (±25)	549 (±26)	0.74	640 (±/)	524 (±29)	/	542 (±53)	509 (±57)	0.74
**CA**	**Dietary phosphorus intake (mg/d)**											
	**Breastfed**			**Formula**			**Mixed**			**No milk**		
	**Early**	**Late**	**p-adj.**	**Early**	**Late**	**p-adj.**	**Early**	**Late**	**p-adj.**	**Early**	**Late**	**p-adj.**
	**Mean ± SE**	**Mean ± SE**		**Mean ± SE**	**Mean ± SE**		**Mean ± SE**	**Mean ± SE**		**Mean ± SE**	**Mean ± SE**	
**6 weeks**	171 (±23)	139 (±11)	0.24	273 (±12)	253 (±19)	0.53	224 (±10)	230 (±24)	0.81	/		
**12 weeks**	136 (±9)	123 (±7)	0.12	293 (±11)	258 (±16)	0.20	199 (±13)	211 (±13)	0.69			
**6 months**	261 (±19)	194 (±15)	**0.04**	381 (±16)	323 (±19)	0.07	403 (±49)	262 (±17)	**0.04**			
**9 months**	335 (±30)	349 (±33)	0.81	459 (±17)	458 (±24)	0.73	608 (±/)	463 (±204)	/	627 (±30)	405 (±101)	/
**12 months**	434 (±72)	517 (±41)	/	548 (±25)	507 (±27)	0.46	445 (±/)	481 (±119)	/	594 (±43)	501 (±42)	0.26
**CA**	**Dietary zinc intake (mg/d)**											
	**Breastfed**			**Formula**			**Mixed**			**No milk**		
	**Early**	**Late**	**p-adj.**	**Early**	**Late**	**p-adj.**	**Early**	**Late**	**p-adj.**	**Early**	**Late**	**p-adj.**
	**Mean ± SE**	**Mean ± SE**		**Mean ± SE**	**Mean ± SE**		**Mean ± SE**	**Mean ± SE**		**Mean ± SE**	**Mean ± SE**	
**6 weeks**	2.04 (±0.37)	1.51 (±0.19)	0.23	4.51 (±0.22)	3.83 (±0.28)	0.18	3.45 (±0.31)	3.11 (±0.55)	0.71	/		
**12 weeks**	1.28 (±0.15)	1.14 (±0.12)	0.12	4.47 (±0.17)	3.90 (±0.20)	0.11	3.08 (±0.26)	2.74 (±0.29)	0.56			
**6 months**	2.14 (±0.16)	1.70 (±0.16)	0.12	4.78 (±0.18)	4.07 (±0.20)	0.11	4.37 (±0.53)	3.42 (±0.35)	0.23			
**9 months**	2.51 (±0.21)	2.74 (±0.23)	0.61	4.93 (±0.14)	4.68 (±0.21)	0.23	3.75 (±/)	5.76 (±2.29)	/	4.42 (±0.72)	3.22 (±0.74)	/
**12 months**	3.04 (±0.45)	4.43 (±0.32)	/	5.27 (±0.18)	5.19 (±0.22)	0.83	6.07 (±/)	4.83 (±1.01)	/	4.29 (±0.30)	4.18 (±0.41)	0.83

CA: corrected age; SE: standard error. p-adj. <0.05 were considered statistically significant and marked bold. No milk: infants that received neither human milk nor infant formula.
